# DUBR non-coding RNA regulates gene expression by affecting AP-1 enhancer accessibility

**DOI:** 10.1007/s10142-025-01582-5

**Published:** 2025-03-21

**Authors:** Simone D. Hall, Khoa Tran, Jonathan Zhu, Tong Su, Colleen A. McHugh

**Affiliations:** Department of Chemistry and Biochemistry, University of California San Diego, Urey Hall Room 6213, 9500 Gilman Drive MC 0314, La Jolla, CA 92093 USA

**Keywords:** DUBR, NuRD complex, HCT116, AP-1, RAP-MS

## Abstract

Non-coding RNAs (ncRNAs) are finely tuned cellular regulators important for human cell growth and cancer progression. DUBR (*Dppa2* upstream binding RNA, also known as linc00883) is a nuclear ncRNA first discovered in mice for its role in regulating myoblast differentiation through interactions with chromatin and DNA methyltransferases. High expression levels of this ncRNA are predictive of poor patient outcome in colon adenocarcinoma, suggesting that DUBR may be involved in controlling cancer growth. To elucidate its function, we used RAP-MS and RNA immunoprecipitation techniques which revealed its interaction with epigenetic maintenance proteins in the human colon cancer cell line HCT116. Further, ATAC-seq and RNA-seq were used to address its function in regulating the epigenome and transcriptome of HCT116 cells. Here we report that DUBR is a regulator of human colon cancer cell line HCT116 survival. Additionally, we find that the ncRNA DUBR regulates AP-1 transcription factor binding site accessibility at enhancers of genes involved in differentiation and morphogenesis through interactions with epigenetic proteins such as NuRD complex members HDAC1 and CHD4.

## Introduction

The epigenome, consisting of chemical modifications to DNA or histone proteins, regulates the accessibility of chromatin to transcription factors and RNA polymerases required for gene expression in human cells. While proteins are foundational modulators of the epigenome, non-coding RNAs (ncRNAs) can also affect chromatin dynamics directly and indirectly. Proposed regulatory mechanisms of ncRNAs in the epigenome include chromatin imprinting, genome organization and directing the localization and function of epigenetic proteins (reviewed in (Statello et al. 2021). However, potential functional roles and mechanisms of regulation remain unknown for the vast majority of ncRNA in the human transcriptome.

Observations of widespread binding of RNA to chromatin associated proteins (Hendrickson et al. 2016) suggest that these functions could be a general mechanism of gene expression regulation through interaction with epigenetic proteins. For example, ncRNAs have been shown to bind and direct epigenetic proteins that regulate histone modifications. As larger protein complexes are typical in this type of regulation, ncRNAs can act as scaffolds and directors for these molecular machines. One such regulatory complex is the nucleosome remodeling and deacetylase (NuRD) complex, where the chromodomain helicase DNA binding protein 4 (CHD4) is responsible for histone remodeling and associated histone deacetylases (HDACs) are responsible for removing H3K27ac acetylation marks. Recruitment and targeting of the NuRD complex can be achieved by ncRNA interactions, such as in the case of the multifunctional gene product PAPAS RNA at ribosomal RNA genes (Zhao et al. 2018).

Additionally, ncRNAs can also modulate the activity of DNA methyltransferases which are responsible for transferring methyl additions to cytosines within the symmetrical dinucleotide CpG. These proteins are categorized as functioning to maintain (DNMT1) or change (DNMT3a and DNMT3b) methylation patterns, repressing gene expression. RNA binding to DNMT1 is widespread and able to both direct (Jones et al. 2021) and inhibit (Di Ruscio et al. 2013) DNMT1 function under different circumstances.

In mice, the non-coding RNA Dubr (*Dppa2* upstream binding RNA) controls myoblast differentiation by directing the function of Dnmt1, Dnmt3a, and Dnmt3b to silence the transcription factor *Dppa2* (Wang et al. 2015). The human DUBR transcript has been proposed to affect a range of cellular processes (Núñez-Martínez et al. 2025; Peralta-Alvarez et al. 2024; Stojic et al. 2020; Yin et al. 2021), though the function of DUBR in regulating genome-wide epigenetic modifications through protein interactions has not yet been evaluated in humans. Therefore, we investigated the mechanism of DUBR activity in epigenetic regulation by using a mass spectrometry approach to discover specific interactions between DUBR and nuclear proteins. We also characterized the cell death phenotype resulting from DUBR knockdown in HCT116 human colon carcinoma cells, A549 lung adenocarcinoma cells and HEK-293 embryonic kidney cells, to expand our understanding of the role of DUBR in regulating survival in different human cell lines.

## Results

### DUBR is a conserved ncRNA that is predictive of poor patient outcome in human colon adenocarcinoma

**Fig. 1:**
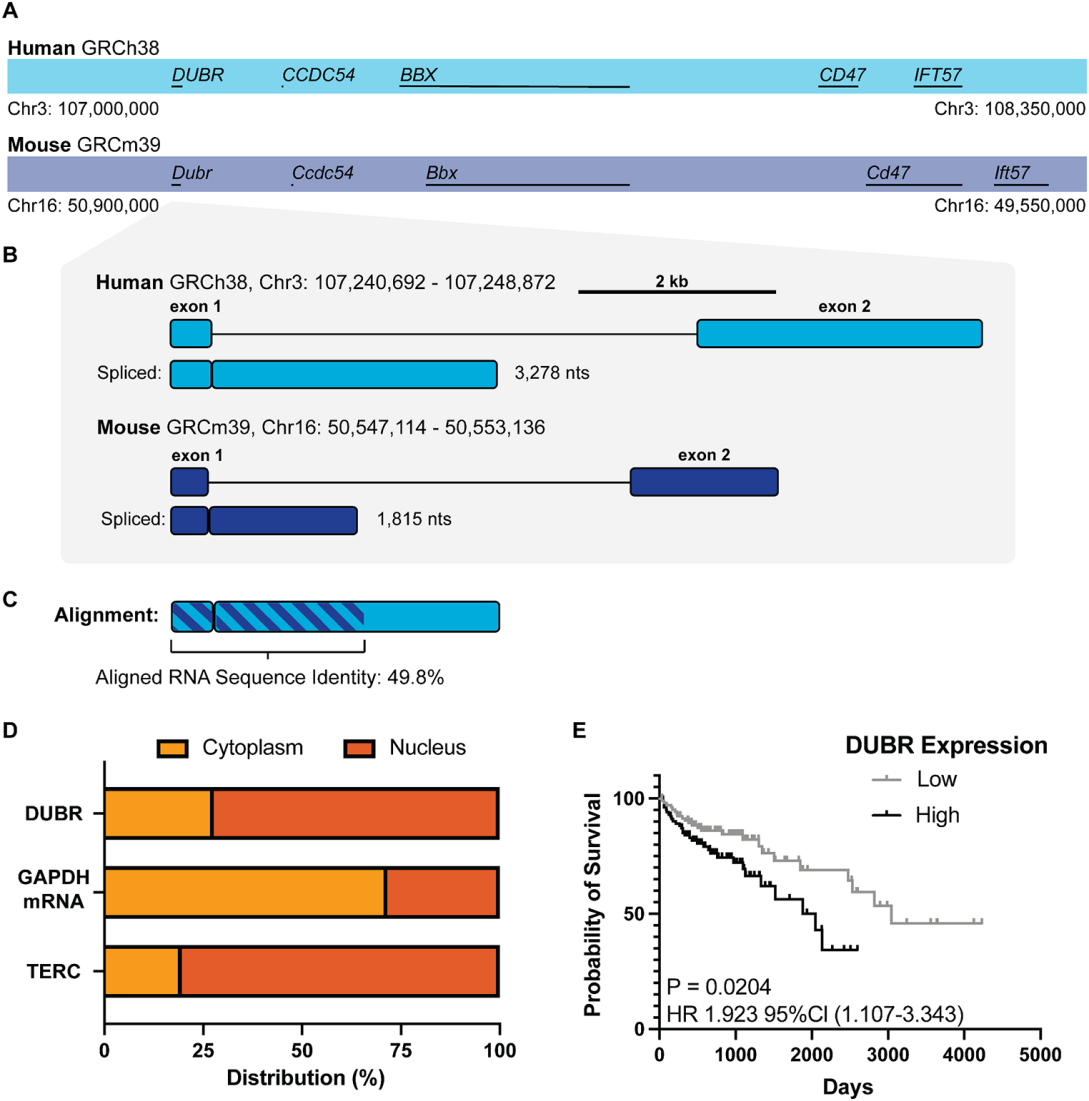
DUBR is a conserved ncRNA that is predictive of poor patient outcome in human colon adenocarcinoma. (**A**) Chromosomal synteny of human *DUBR* (light blue) and mouse *Dubr* (navy). (**B**) Genomic architecture and spliced RNA comparison of human DUBR (NCBI RefSeq: NR_028301.1) and mouse Dubr (NCBI RefSeq: NR_028300.1). (**C**) Pair-wise sequence alignment of mouse Dubr against human DUBR RNA transcript. (**D**) Cellular localization of DUBR RNA compared to nuclear control TERC telomerase RNA and cytoplasmic control *GAPDH* mRNA in HCT116. (**E**) Kaplan-Meier survival curve for high (*N* = 110) and low (*N* = 110) DUBR expression in the TCGA colon adenocarcinoma data set

DUBR is a spliced, intergenic non-coding RNA that shows conserved transcript sequence and synteny between mouse and human (Fig. 1A). Both mouse Dubr and human DUBR are comprised of two exons and contain a conserved GU-AG splice site between exon 1 and exon 2. Mouse Dubr shares around 50% sequence identity with human DUBR by pairwise sequence alignment (Fig. 1B, C). Cellular fractionation and qPCR analysis of DUBR show that the transcript is primarily nuclear localized, similar to the nuclear control telomerase ncRNA TERC. In cellular fractionation experiments, the *GAPDH* mRNA control was localized primarily in the cytoplasm, as expected (Fig. 1D). In addition to the role of Dubr in mouse myoblast development, the human DUBR transcript has been suggested to play a variety of roles in cells (Núñez-Martínez et al. 2025; Peralta-Alvarez et al. 2024; Stojic et al. 2020; Yin et al. 2021). Analysis of the TCGA colon adenocarcinoma dataset accessed using OncoLnc (Anaya 2016) shows that high expression of DUBR in patient tumors is predictive of poor patient survival (Fig. 1E).

### DUBR knockdown inhibits HCT116 proliferation and invasion

**Fig. 2:**
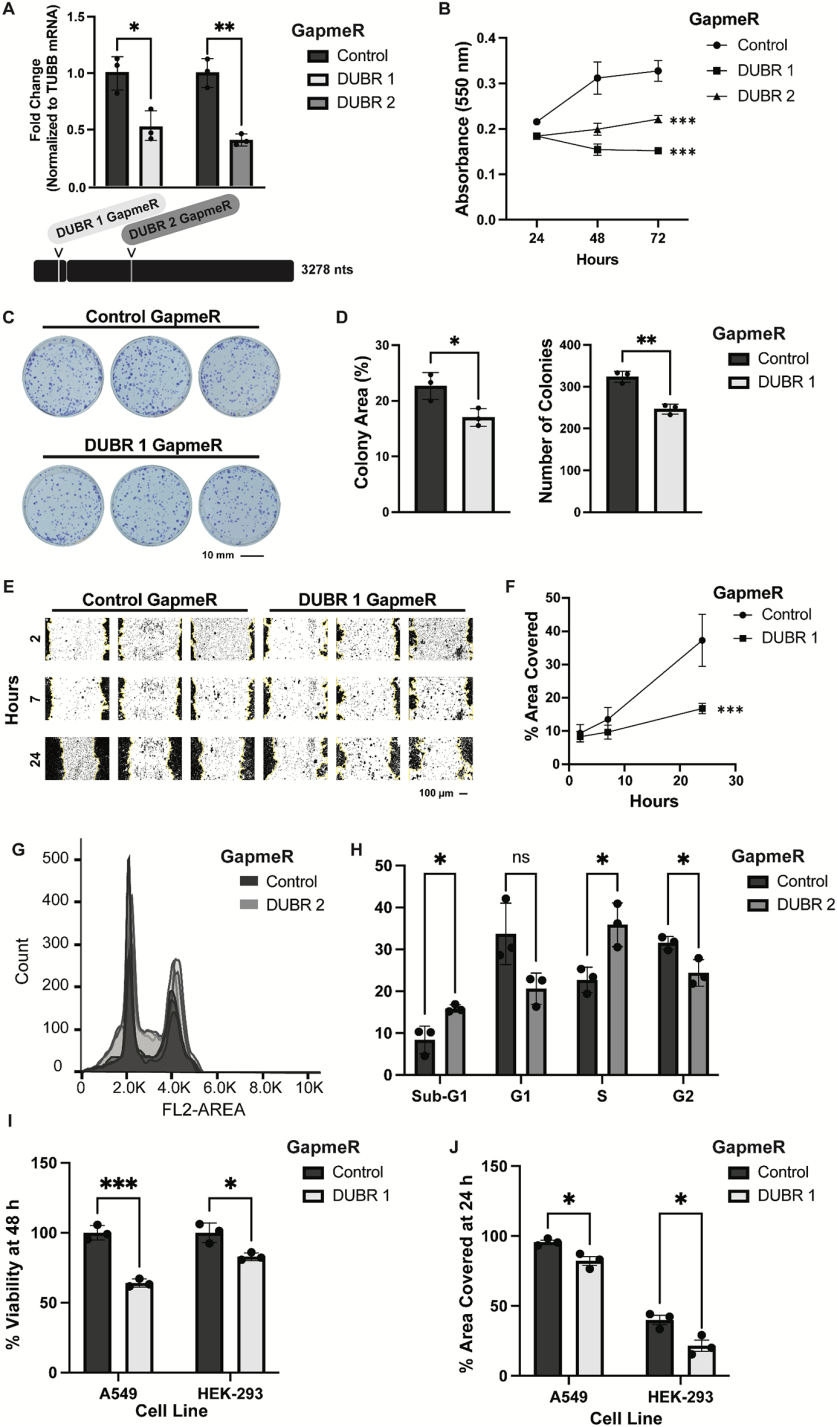
DUBR knockdown causes cell death, reduced invasion and cell cycle changes in HCT116 cells. (**A**) Knockdown efficiencies of 48-hour DUBR 1 and DUBR 2 GapmeR treatment in HCT116 and their target location on exon 1 and exon 2 of DUBR, respectively. (**B**) Growth effect of 24-, 48- and 72-hour GapmeR-mediated DUBR knockdown in HCT116 assessed by MTT assay. (**C**) Colony formation assay of control and DUBR 1 GapmeR treated HCT116 cells. (**D**) Quantification of colony area % and colony number of colony formation assay. (**E**) Scratch assay of control and DUBR 1 GapmeR treated HCT116 cells. (**F**) Quantification of % area covered in scratch assay. (**G**) Cell cycle analysis using flow cytometry after staining with propidium iodide (PI) in DUBR 2 and control GapmeR treated HCT116 cells. (**H**) Quantification of cell cycle distribution changes from flow cytometry data. (**I**) Cell viability in human lung adenocarcinoma cell line A549 and human embryonic kidney cell line HEK-293 after 48-hour treatment with control or DUBR 1 GapmeR assessed by MTT assay. (**J**) Scratch assay results at 24 h in A549 and HEK-293 cells treated with control or DUBR 1 GapmeR. All experiments performed *N* = 3. Error bars represent mean ± SD. *P ≤ 0.05; **P ≤ 0.01; ***P ≤ 0.001 by two-tailed Student’s t-test

To evaluate whether DUBR non-coding RNA contributes to proliferation in the human colon cancer cell line HCT116, two GapmeR antisense oligonucleotide sequences were designed to knock down DUBR RNA expression. Transfection with either of the GapmeRs achieved efficient DUBR knockdown (Fig. 2A). GapmeR-mediated knockdown was selected over other deletion or genetic mutation methods to ensure that results were dependent on specific depletion of the RNA transcript, and not due to changes in transcriptional regulation or genetic elements encoded at the locus. Additionally, GapmeRs are ideal for targeting nuclear RNA transcripts because they can achieve more efficient knockdown than siRNA or shRNA treatments (Maranon and Wilusz 2020).

In the human colon cancer cell line HCT116, each of the DUBR GapmeRs were able to significantly inhibit cell growth after 24-, 48- and 72-hour treatments (Fig. 2B). Colony formation assay shows that treatment with DUBR 1 GapmeR reduces colony area and number of colonies when compared to control GapmeR treatment (Fig. 2C, D). Furthermore, the scratch assay test shows that HCT116 cell migration is also significantly decreased after DUBR 1 GapmeR treatment (Fig. 2E, F). The results indicate that expression of the ncRNA DUBR promotes HCT116 proliferation and migration. These data agree with the Kaplan-Meier survival curve analysis, where high expression of DUBR ncRNA in the TCGA colon adenocarcinoma dataset was predictive of poor patient outcome.

Additionally, flow cytometry was used to investigate whether DUBR is involved in cell cycle regulation as a previous screen identified that it was important for cell division (Stojic et al. 2020). After 48 h of treatment with DUBR 2 GapmeR, there were significant changes in cell cycle distribution with an increase of DUBR KD cells in the S-phase and a decrease of cells in the G2-phase. Furthermore, there was an increase in the population of cells in sub-G1 which are indicative of apoptotic cell death (Fig. 2G, H).

To see if DUBR had similar phenotypic effects in other cell lines, the cell viability assay and scratch assay were repeated in the human lung adenocarcinoma cell line A549 and non-cancerous human embryonic kidney cell line HEK-293. After 48 h of DUBR 1 GapmeR treatment there was a significant decrease in cell viability in both cell lines (Fig. 2I). Similarly, 24-hours post scratch, both A549 and HEK-293 cells pre-treated with DUBR 1 GapmeR for 48 h had less mobility than control GapmeR treated cells (Fig. 2J). These results indicate that DUBR ncRNA knockdown has similar phenotypic effects in multiple human cell lines.

### Endogenous DUBR transcript binds directly to epigenetic regulatory proteins

**Fig. 3:**
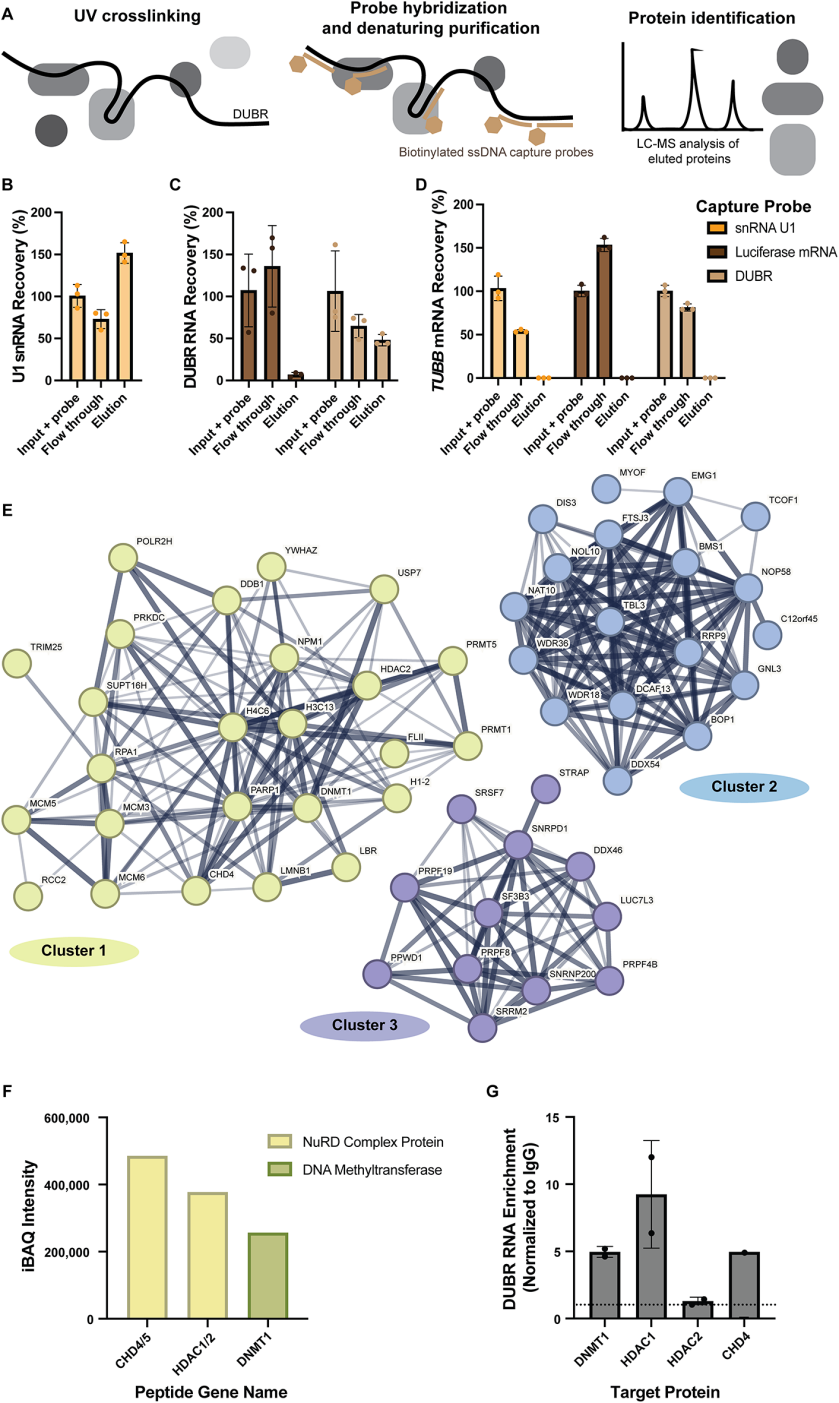
Endogenous DUBR RNA binds epigenetic proteins. (**A**) RNA antisense purification with mass spectrometry (RAP-MS) method overview. RNA recoveries of (**B**) U1 snRNA by U1 capture probes, (**C**) DUBR RNA by DUBR RNA and luciferase mRNA capture probes, (**D**) *TUBB* mRNA by U1 snRNA, DUBR RNA and luciferase mRNA capture probes. (**E**) STRING cluster analysis of unique DUBR RAP-MS nuclear protein hits shows proteins grouped by interaction networks. (**F**) iBAQ values for select proteins in Cluster 1. (**G**) Compiled RNA immunoprecipitation (RIP) data for DUBR enrichment in epigenetic regulator protein captures. Dashed line represents DUBR enrichment cut-off of 1-fold. RIP replicates: DNMT1, *N* = 2; HDAC1, *N* = 2; HDAC2, *N* = 2; CHD4, *N* = 1

ncRNAs function in cells through interactions with other macromolecules including other RNAs, DNA and proteins. To gain insight into the molecular mechanisms of this RNA we used the RNA antisense purification with mass spectrometry (RAP-MS) technique. This approach was previously used successfully to identify direct binding partners of the ncRNA XIST (McHugh et al. 2015) and has since been adapted and optimized by our group for use in human cancer cells (Trang et al. 2023). Briefly, RAP-MS identifies direct and specific protein binding partners of an RNA by capturing UV_254nm_-crosslinked RNA-protein complexes from human cells with complementary biotinylated ssDNA capture probes on streptavidin-coated magnetic beads. Endogenous RNA-protein complexes are subsequently enriched and isolated through highly denaturing washes and identified by mass spectrometry (Fig. 3A). Negative control probes for the RAP-MS experiment were biotinylated ssDNA capture probes complementary to firefly luciferase (*Luc*) mRNA. These were used to filter and remove proteins that bound non-specifically to ssDNA or are nonspecific interactors with biotin or streptavidin. Capture probes targeting the small nuclear RNA (snRNA) U1 were used as additional controls to identify common RNA binding proteins and splicing factors. Capture probes against snRNA U1 and DUBR RNA were tested for recovery of target RNA, while lack of recovery of DUBR RNA was tested for negative control luciferase mRNA capture probes (Fig. 3B, C). Additionally, the specificity of the capture probes was tested by looking for minimal non-specific binding of *TUBB* mRNA (Fig. 3D).

To determine if DUBR was interacting with particular types of proteins, we used STRING analysis (Szklarczyk et al. 2023) which uses interactome data to group proteins. Because DUBR is localized to the nucleus, STRING analysis was performed on proteins that (1) had at least two unique peptides identified in the DUBR RAP-MS elution, (2) were not hits in any of the control samples U1 snRNA and luciferase mRNA, and (3) had evidence for nuclear localization based on the Protein Atlas database (Pontén et al. 2008). This analysis revealed three clusters of proteins (Fig. 3E). Cluster 1, the largest cluster, contained chromatin associated proteins with top gene ontology (GO) biological processes terms associated with epigenetic regulation or DNA damage repair and cellular component terms with similar functions (Table 1). Cluster 2 and 3 contain RNA binding proteins involved in RNA modification and RNA splicing, respectively (Tables 2 and 3).

**Table 1:** Cluster 1 gene ontology

Cellular Component	Enrichment	*P*-value
MCM complex	2.47	4.64e^− 5^
CMG complex	2.33	8.85e^− 5^
Methylosome	2.12	0.0114
Nuclear replisome	2.08	0.0126
NuRD Complex	2.02	0.0154
**Biological Process**		
Histone H4-R3 methylation	2.42	0.031
Maintenance of DNA methylation	2.35	0.057
Double-strand break repair via break-induced replication	2.29	0.00047
Regulation of DNA-templated DNA replication initiation	2.20	0.00078
DNA unwinding involved in DNA replication	2.16	4.62e^− 5^

**Table 2:** Cluster 2 gene ontology

Cellular Component	Enrichment	*P*-value
Box C/D RNP complex	2.50	0.0041
Small-subunit processome	2.29	4.97e^− 12^
90 S preribosome	2.19	5.87e^− 6^
Preribosome	2.16	1.26e^− 16^
Preribosome, large subunit precursor	2.02	0.0237
**Biological Process**		
Endonucleolytic cleavage of tricistronic rRNA transcript	2.31	0.00046
Maturation of SSU-rRNA from tricistronic rRNA transcript	2.05	4.84e^− 5^
Maturation of SSU-rRNA	2.00	1.69e^− 6^
rRNA modification	1.96	0.0036
Ribosomal small subunit biogenesis	1.93	1.07e^− 7^

**Table 3:** Cluster 3 gene ontology

Cellular Component	Enrichment	*P*-value
U2-type catalytic step 1 spliceosome	2.58	1.41e^− 5^
U5 snRNP	2.41	3.37e^− 5^
U2-type catalytic step 2 spliceosome	2.34	6.17e^− 7^
U2 snRNP	2.29	6.03e^− 5^
SMN-Sm protein complex	2.29	0.0042
**Biological Process**		
Spliceosomal tri-snRNP complex assembly	2.40	0.0180
U2-type prespliceosome assembly	2.31	0.00033
Spliceosomal snRNP assembly	2.23	7.52e^− 6^
mRNA cis splicing, via spliceosome	2.15	0.0481
Spliceosomal complex assembly	2.11	5.09 e^− 9^

We further examined the interactions of DUBR with proteins in Cluster 1 because of the potential for conservation of epigenetic function between mouse and human. The mouse ortholog of DUBR has been shown to regulate Dnmt1 function in mouse myogenesis (Wang et al. 2015). DNMT1, HDAC1, HDAC2 and CHD4 were selected for additional validation because of their roles in epigenetics and direct RNA binding ability (Hendrickson et al. 2016). These proteins can also interact as a repressive super complex in colon cancer (Cai et al. 2014). The iBAQ intensities for these proteins are shown in Fig. 3F. RNA immunoprecipitation (RIP) assays confirmed that DUBR ncRNA was enriched in each of the DNMT1, HDAC1 and CHD4 captures, but was not enriched in HDAC2 captures, validating the results from RAP-MS (Fig. 3G).

### DUBR knockdown results in global changes in chromatin accessibility

**Fig. 4:**
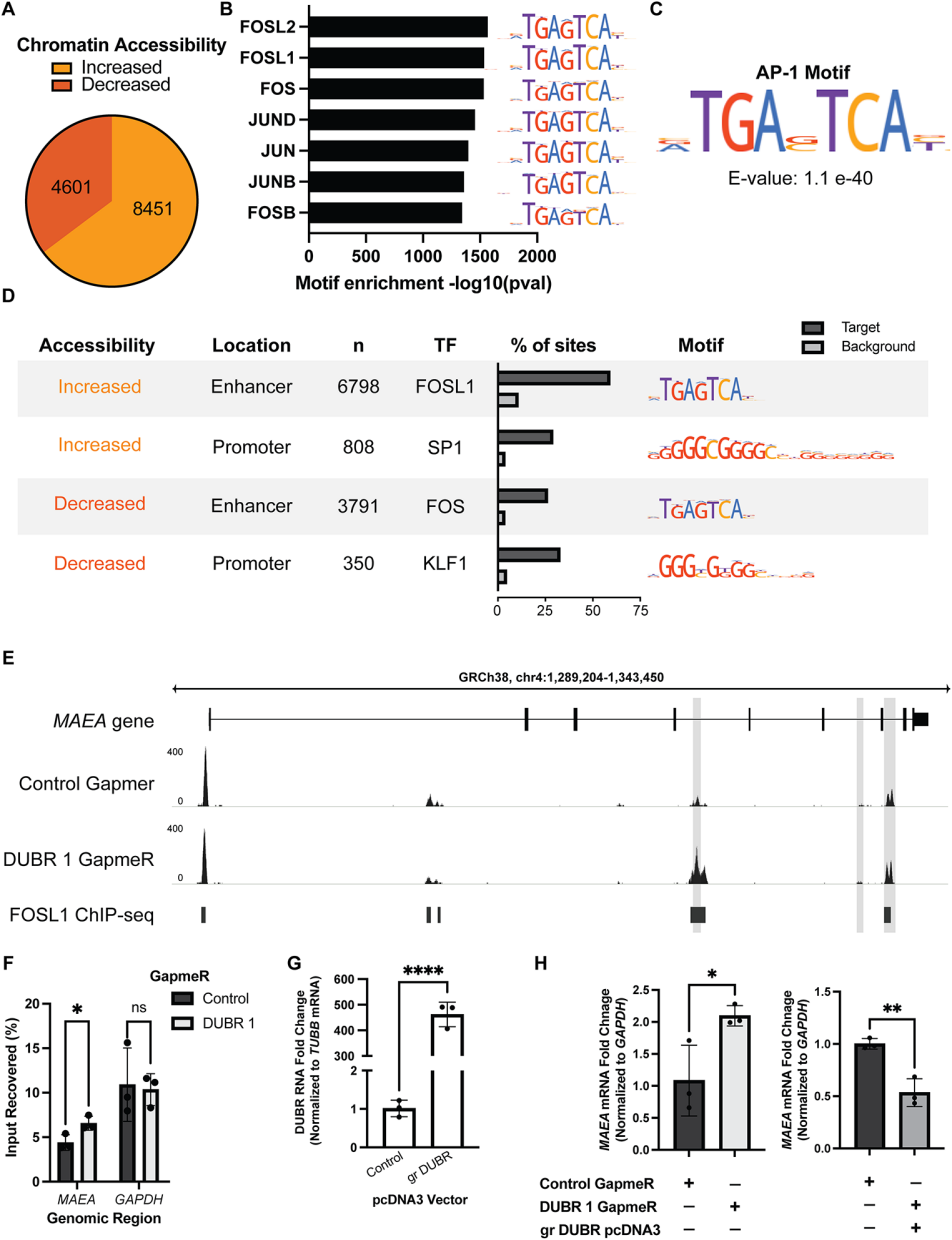
DUBR knockdown results in global changes in chromatin accessibility. (**A**) Distribution of differentially increased and decreased accessible peaks after 48-hour knockdown with DUBR 1 GapmeR compared to control GapmeR. (**B**) MEME SEA transcription factor motif enrichment in all differentially accessible peaks. (**C**) MEME STREME top discovered *de novo* motif in all differentially accessible peaks. (**D**) Top enriched transcription factor, respective motif, and percent of sites containing motif compared to background using MEME SEA analysis of differentially accessible peak subsets. (**E**) ATAC-seq peaks at *MAEA* gene locus showing multiple sites with increased chromatin accessibility in DUBR knockdown cells (light grey bar overlay). (**F**) Recovery of *MAEA* enhancer DNA compared to control *GAPDH* enhancer DNA for H3K27ac ChIP-qPCR in DUBR and control knockdown (KD) cells. (**G**) Overexpression efficiency of pcDNA3 GapmeR-resistant (gr) DUBR vector. (**H**) Fold change in *MAEA* mRNA expression after DUBR KD vs. control KD and DUBR KD with (gr) DUBR overexpression vs. control KD. *N* = 3. Error bars represent mean ± SD. ns *P* > 0.05; *P ≤ 0.05; **P ≤ 0.01; ***P ≤ 0.001; ****P ≤ 0.0001 by two-tailed Student’s t-test

To address the role of DUBR in epigenome maintenance, chromatin accessibility changes after DUBR knockdown were monitored by assay for transposase-accessible chromatin using sequencing (ATAC-seq). Performed in triplicate and compared against control knockdown HCT116 cells, ATAC-seq results showed global changes, with a higher proportion of peaks increasing in accessibility in DUBR knockdown cells (Fig. 4A). Using the MEME suite (Bailey et al. 2015), SEA analysis identified the family of AP-1 transcription factors as being the most highly enriched across all differentially accessible peaks (Fig. 4B). Additionally, STREME analysis identified the AP-1 consensus binding motif as the most enriched *de novo* motif (Fig. 4C). AP-1 transcription factors have a wide range of functions including maintaining somatic cell identity and inhibiting reprogramming (Markov et al. 2021). We then classified differentially accessible peaks based on location to transcription start site (TSS) as promoter (< 1 kb to TSS) or enhancer (> 1 kb to TSS). SEA analysis on these populations shows clear differences in the enriched motifs. Differentially accessible peaks in promoters, both increased and decreased in accessibility, show highest enrichment for motifs with high C/G content, while in enhancers the AP-1 transcription factor motifs are still the most highly enriched (Fig. 4D). In the enhancer regions with increased accessibility, we see that over 60% of these peaks have the FOSL1 motif as compared to the decreased accessible peaks for which just under 30% have the FOS motif.

Enhancer accessibility is dependent upon many different histone modifications, of which H3K27ac is an important mark for maintaining open chromatin. The NuRD complex, as a repressor, removes this mark to decrease accessibility at enhancers. Hypothesizing that DUBR knockdown could interfere with the NuRD complexes function in silencing enhancers and thus leading to the increased accessibility and mRNA expression changes, we looked at the change in H3K27ac levels at one of the most significantly changed peaks which is an intragenic enhancer for the *MAEA* gene locus, which encodes for a E3 ubiquitin ligase that mediates the attachment of erythroblasts to macrophages (Fig. 4E). Performing ChIP-qPCR against this histone modification, upon DUBR knockdown there is an increase in H3K27ac as predicted (Fig. 4F). To see if this results in a change in *MAEA* mRNA expression, we performed qPCR analysis and observed an increase in *MAEA* mRNA expression upon DUBR 1 GapmeR treatment. Furthermore, complementation by overexpression of a GapmeR-resistant DUBR RNA transcript (gr DUBR, overexpression confirmed in Fig. 4G) leads to reduced *MAEA* mRNA expression, indicating that the DUBR transcript is directly responsible for regulating *MAEA* (Fig. 4H).

### DUBR knockdown results in global changes in mRNA expression

**Fig. 5:**
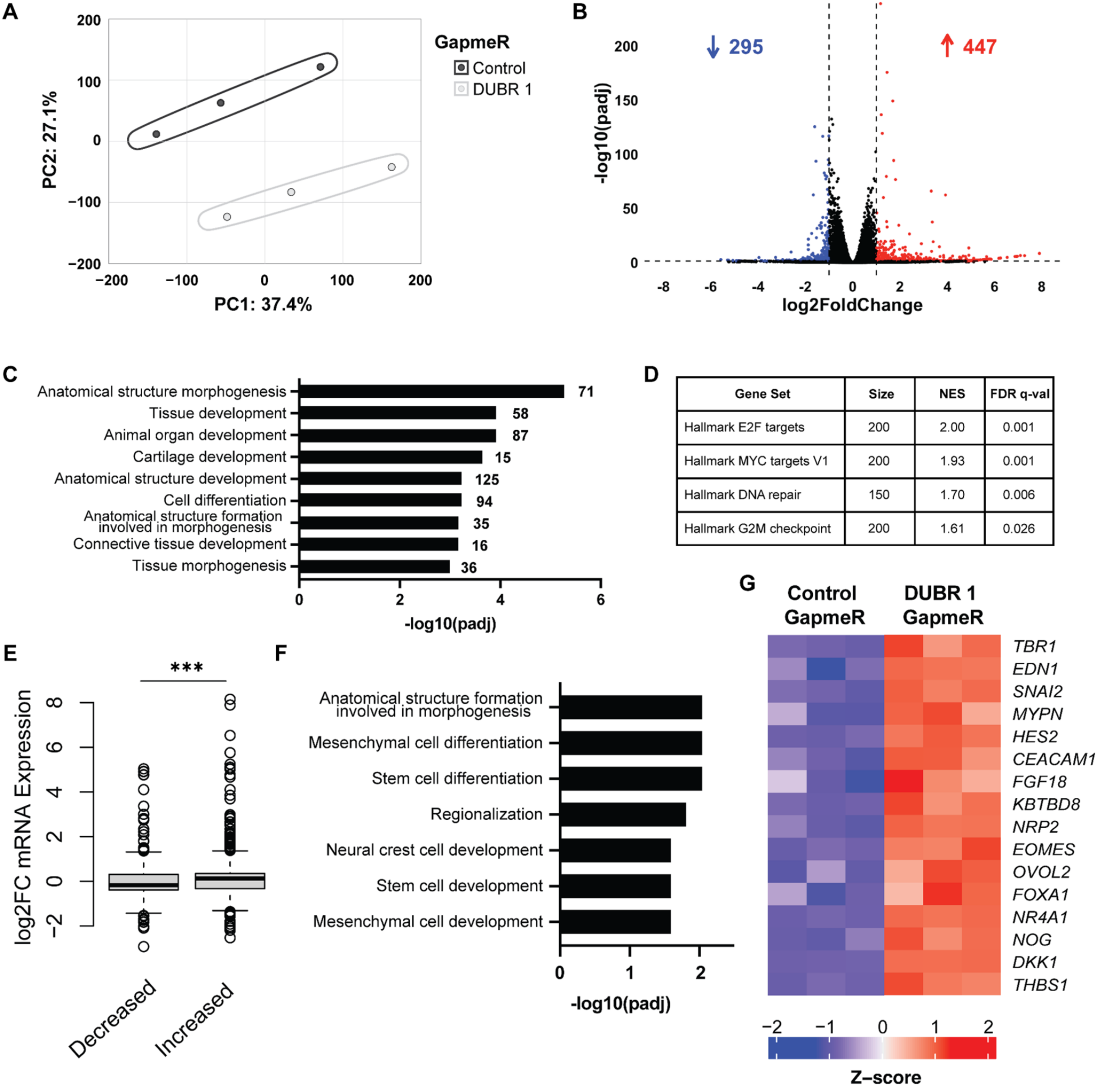
DUBR knockdown results in global changes in mRNA expression. (**A**) PCA analysis of control and DUBR 1 GapmeR replicates. (**B**) Volcano plot of mRNA expression after 48-hour DUBR 1 GapmeR knockdown in HCT116. Significantly upregulated genes (red) are defined as having log2FC > 1 and P-value < 0.05 and significantly downregulated genes (blue) are defined as having log2FC < -1 and P-value < 0.05. (**C**) Top enriched gene ontology biological processes for significantly upregulated genes after DUBR knockdown. (**D**) GSEA enriched hallmark gene sets. (**E**) Log2FC of significantly different mRNA expression plotted by enhancer chromatin accessibility. (**F**) Top enriched gene ontology of biological processes for significantly upregulated genes with matched enhancer that are significantly increased in accessibility after DUBR knockdown. (**G**) Heatmap of RNA expression of genes in gene-enhancer pairs that make up top gene ontology biological processes. ***P ≤ 0.001 by two-tailed Student’s t-test

Because of the global changes in chromatin accessibility after DUBR knockdown, we chose to do RNA sequencing (RNA-seq) after 48-hour DUBR 1 GapmeR treatment. RNA-seq was performed in biological triplicate to enable statistical analysis. PCA analysis showed clustering of control and experimental samples, indicating high concordance among the sample sets (Fig. 5A). Using a log2-fold change cut-off value of ± 1 and P-value < 0.05, we see 295 genes decreasing in expression and 447 genes increasing in expression between control and DUBR 1 knockdown cells (Fig. 5B). Interestingly, in contrast to mouse Dubr which was seen to regulate expression in cis (Wang et al. 2015), DAVID analysis (Sherman et al. 2022) of expression change by genome locations did not identify any specific genomic regions with significantly altered gene expression, including the *DUBR* genomic locus.

Because DUBR RNA interacts with repressive maintenance epigenetic proteins, we next used GO term analysis to identify any trends in the differentially regulated gene set after DUBR knockdown. While there were no significantly enriched GO-term pathways in the 295 down regulated genes, analysis of the upregulated gene set highlighted an enrichment in genes associated with morphogenesis and development biological processes (Fig. 5C), supporting the hypothesis that DUBR promotes maintenance of somatic cell identity. Furthermore, GSEA analysis of the RNA-seq data showed significant enrichment of several hallmark datasets (Fig. 5D). These include transcription factor targets involved in S-phase progression and cell cycle gene sets. These results show that our RNA sequencing data reveal insight into the cell death and cell cycle arrest phenotypes observed in the cellular assays.

To identify global overlap between changes in chromatin accessibility and gene expression, we used the activity-by-contact method (Fulco et al. 2019) where we used publicly available HCT116 Hi-C data (Du et al. 2021), ENCODE H3K27ac ChIP-seq data and ATAC-seq data (ENCODE Project Consortium 2012) to define and pair enhancers to genes. By then comparing enhancer-gene interactions of the differentially accessible chromatin peaks and RNA-seq data we see that increased accessibility is correlated with increased RNA expression at affected loci, and vice versa, as expected (Fig. 5E). GO term analysis of the subset of genes that have increased expression and increased enhancer accessibility after DUBR knockdown reveals biological processes similar to those found in the set of all overexpressed genes (Fig. 5F), indicating that this core subset is responsible for the trends we see overall. Many of the genes in this subset are known to be regulated by AP-1 such as *EDN1* (Kawana et al. 1995), *SNAI2* (Zhang et al. 2021), and *DKK1* (Shao et al. 2024) (Fig. 5G).

## Discussion

We investigated the function of a conserved ncRNA in regulating cell growth and epigenetic maintenance. We find that the human ncRNA DUBR is required for growth in the colon cancer cell line HCT116. Loss of DUBR expression in HCT116 leads to changes in chromatin structure and an upregulation of genes involved in differentiation and morphogenesis, similar to the phenotypes observed in previous studies (Núñez-Martínez et al. 2025). The mouse ortholog ncRNA Dubr was previously shown to localize to the nucleus and interact with Dnmt1 to silence nearby genes. Based on these findings, we hypothesized that DUBR might similarly regulate human gene expression through nuclear interactions with epigenetic regulatory proteins. We identified several novel protein interaction partners of DUBR in HCT116 cells. Using a highly denaturing affinity purification method, RAP-MS, we find that DUBR ncRNA binds directly to epigenetic regulatory proteins DNMT1, HDAC1 and CHD4. DNMT1 is a methyltransferase responsible for maintaining methylation on the nascent DNA strand after replication during the S-phase (Petryk et al. 2021). Similarly, CHD4 and HDAC1 participate as part of the nucleosome remodeling and deacetylase (NuRD) complex which is a repressive epigenetic protein complex. The NuRD complex removes H3K27ac marks from open chromatin, to regulate DNA accessibility for transcription and facilitate tissue-specific gene expression patterns (Reid et al. 2023). Both DNMT1 and the NuRD complex are repressive and work to maintain the epigenome of somatic cells (Cai et al. 2014). The direct interactions between DUBR and the epigenetic regulatory factors identified in these experiments indicate a likely function of DUBR in gene repression.

Because we identified DNMT1 and other epigenetic regulatory factors as direct binding partners of DUBR ncRNA in HCT116 cells, we investigated whether loss of DUBR ncRNA resulted in changes in chromatin accessibility and gene expression. Upon DUBR knockdown, we observed a significant change in ATAC-seq signal at loci across the genome, with a concomitant increase in mRNA expression of genes involved in differentiation and morphogenesis. Loss of DUBR ncRNA leads to changes in chromatin accessibility focused around AP-1 binding sites in enhancers, which may be maintained by HDAC1 and CHD4 as part of the NuRD complex regulating H3K27ac marks on chromatin. The NuRD complex has been previously reported to repress AP-1 binding sites at enhancers and affect chromatin accessibility in human keratocytes (Shibata et al. 2020). In our work, enhancer-gene mapping reveals DUBR mediated repression of developmental genes through maintaining low chromatin accessibility at their AP-1 enhancers. Complementation of DUBR knockdown with DUBR ncRNA overexpression confirmed the direct role of DUBR ncRNA in affecting chromatin accessibility and gene expression in HCT116 cells. Interestingly, in human hematopoietic cell line K562, a similar function was discovered when DUBR knockout resulted in a global increase in H3K27ac and affected the expression of genes important for hematopoietic differentiation (Núñez-Martínez et al. 2025).

From these data, we conclude that DUBR functions in maintenance of epigenetic marks in HCT116 cells. Similar to the role of Dubr ncRNA in mouse, human DUBR ncRNA binds directly to repressive epigenetic proteins to maintain cell identity. When DUBR expression is decreased, HCT116 cell viability is decreased as well. Previous work showed that DUBR is involved in protection against DNA damage, and intriguingly, DUBR was identified in a screen for DNA damage protective factors in HeLa cells (Stojic et al. 2020). Additionally, DUBR knockout in the human hematopoietic cell line K562 cells showed similar effects on cell viability, global chromatin accessibility changes, and transcription regulation (Núñez-Martínez et al. 2025). The contribution of DUBR to cell growth and chromatin regulation in mouse and multiple human cell types, in this report and other studies, suggests that this non-coding RNA transcript has an important function in epigenetic regulation.

**Fig. 6:**
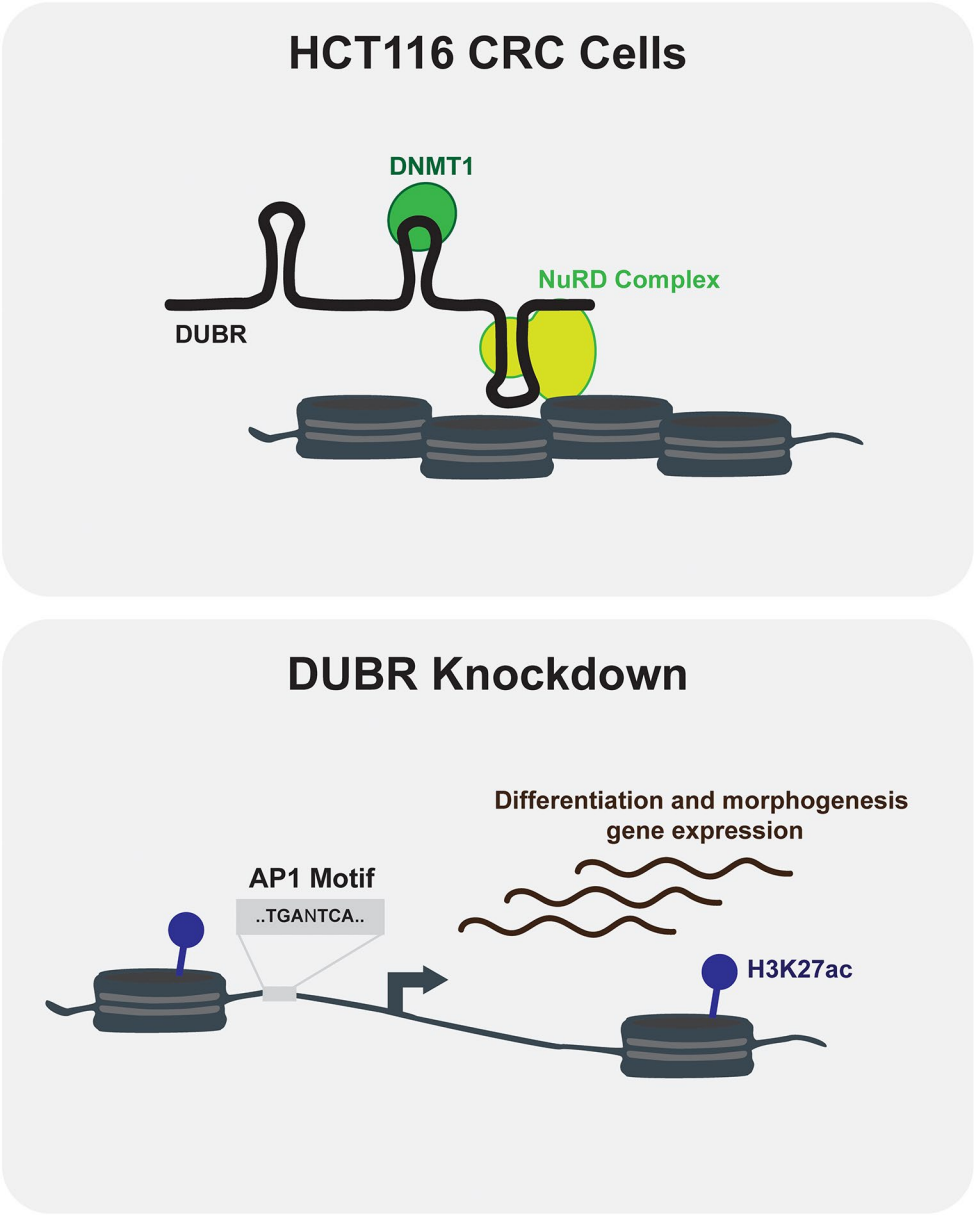
Model of DUBR ncRNA regulation of chromatin accessibility in HCT116 cells

Non-coding RNAs can regulate chromatin structure and gene expression in many ways. Because DUBR interacts with multiple epigenetic regulators it is possible that this ncRNA is acting as a scaffold for the NuRD complex and potentially DNMT1. Additional experiments would be required to determine if DNMT1 contributes to silencing of DUBR regulated genes, since DNA methylation changes were not addressed in the current study. Additionally, determining whether DUBR specificity occurs through direct DNA-RNA interaction, secondary protein interactions, or an alternative method will be an important next step in identifying the molecular mechanisms of DUBR in regulating chromatin accessibility and gene expression. Finally, a similar regulatory function of DUBR was observed in the human hematopoietic cell line K562 (Núñez-Martínez et al. 2025). Based on this work, and our studies of cell death and migration phenotypes in the cell lines A549 and HEK-293, the generality and mechanism of DUBR function warrant further investigation. An overview of the proposed model of DUBR function in HCT116 cells is provided in Fig. 6.

In summary, we find that DUBR has a conserved function in epigenetic regulation in mice and humans. In mice, Dubr was shown to regulate the expression of neighboring genes. In humans, neighboring gene expression is similarly inhibited by DUBR expression, while DUBR also maintains genome-wide silencing of enhancers regulated by AP-1 transcription factor binding. One mechanism by which this genome-wide function of DUBR could occur is through the binding of DUBR to NuRD complex subunits, because we find that direct RNA-protein interactions occur between DUBR and epigenetic regulators DNMT1, CHD4, and HDAC1. Growth control in cancer cells is complex and depends on multiple layers of regulation. If similar interactions occur during human tumorigenesis, then RNA therapeutics targeting the release of DUBR-mediated silencing might potentially be explored as anti-tumor treatments.

## Experimental methods

### Conservation analysis

Human DUBR transcript NR_028301.1 was aligned with mouse Dubr transcript NR_028300.1 using the EMBOSS needle pairwise sequence alignment tool (Madeira et al. 2024). The sequence similarity % was calculated for the % of mouse Dubr sequence that aligned to human DUBR.

### Kaplan-Meier survival analysis

Kaplan-Meier survival analysis was done with COAD TCGA data downloaded using the online tool OncoLnc (Anaya 2016). Top and bottom 25% were used for high and low DUBR expression levels. Data analysis and Kaplan-Meier graph was done using Prism. Log-rank P-value calculated using Mantel-Cox method and Hazard Ratio calculated using Mantel-Haenszel method.

### Cell culture

Human colon carcinoma cell line HCT116 (No. CCL-247, ATCC, USA) cells were cultured in McCoy’s 5 A modified medium (Gibco, USA) supplemented with 10% fetal bovine serum (Gibco) and 1% additional L-Glutamine (Corning, USA).Human lung adenocarcinoma cell line A549 (No. CCL-185, ATCC, USA) and human embryonic kidney cell line HEK293 (No. CRL-1573, ATCC, USA) were cultured in Eagle’s minimum essential medium (Quality Biological, USA) supplemented with 10% fetal bovine serum (Gibco) and 1% additional L-Glutamine (Corning, USA). Cells were grown at 37˚C with 5% CO_2_ and tested monthly for mycoplasma.

### Antisense oligonucleotide (ASO) GapmeR knockdown of DUBR

For DUBR RNA knockdown, cells were seeded and allowed to grow overnight. The next day they were transfected with control (Qiagen, USA; 30300019-2), DUBR 1 (Qiagen; custom design, target sequence: 5’- ACGGAGCAAATCGGAA − 3’) or DUBR 2 (Qiagen; custom design, target sequence: 5’- CTGTTAGACTCATCGA − 3’) GapmeR per Qiagen recommendations using lipofectamine 2000 (Thermo Fisher Scientific, USA) in Opti-MEM I (Gibco) reduced serum medium following the manufacturer’s suggested protocol.

### Plasmid overexpression of GapmeR-resistant (gr) DUBR

The gr DUBR overexpression vector was generated by PCR from HCT116 complementary DNA using locus-specific primers (Table 4) and primers with overhangs scrambling the DUBR 1 GapmeR target sequence. The pcDNA3 backbone was amplified with overhangs complementary to DUBR 5’ and 3’ ends and the three fragments were Gibson cloned using Gibson Assembly Master Mix (E2611, New England Biolabs, USA). Empty pcDNA3 vector was used as a control when checking DUBR overexpression by qPCR.

**Table 4:** PCR primer sequences

Primers	Sequence (5’ − 3’)
*DUBR* cloning	F: ACCCACGCGGCGCAGC
	R: CAATAAATAAACCTTATTTATTATAAGGAATTGGC TTACACAATAATGG
DUBR ncRNA qPCR	F: TGAGGGTTGAAATGGAGAGG
	R: CGATCATATGGGGCATCC
*GAPDH* mRNA qPCR	F: GGGCTCTCCAGAACATCATCC
	R: GTCCACCACTGACACGTTGG
TERC ncRNA qPCR	F: CCCTAACTGAGAAGGGCGTAG
	R: TGCTCTAGAATGAACGGTGGA
*TUBB* mRNA qPCR	F: CCAGACAACTTTGTATTTGGTCAGT
	R: CGTACCACATCCAGGACAGAAT
U1 snRNA qPCR	F: TTACCTGGCAGGGGAGATAC
	R: TCCCACATTTGGGGAAATC
*MAEA* mRNA qPCR	F: AGCTGCCTGGAGTTCAGCCTCA
	R: TCCAGCTGGCTCCCTTCTGCTT
G*APDH* enhancer qPCR	F: CCACATCGCTCAGACACCAT
	R: GCGAACTCACCCGTTGACT
*MAEA* enhancer qPCR	F: TCCTGCAAGCCCTAATTACCTG
	R: TTCAGACAGGTCGTGTGCTC

### Nucleic acid isolation and quantitative PCR (qPCR)

Total RNA was isolated using the RNeasy kit with on-column DNase digestion (Qiagen) following manufacture protocols. 1 µg of purified RNA was reverse transcribed using 10-mer random primers with Superscript III Reverse Transcriptase (18080085, Thermo Fisher Scientific). Quantitative PCR was performed using ROX Reference Dye (Thermo Fisher Scientific) and SYBR Green Dye (Thermo Fisher Scientific) on a QuantStudio™ 3 Real-Time PCR System, 96-well (Thermo Fisher Scientific). Fold expression was calculated using the 2^−ΔΔCt^ method. All primers used can be found in Table 4.

### Cellular fractionation

1 × 10^7^ HCT116 cells were collected by trypsinization and resuspended in 1 mL hypotonic lysis buffer (10 mM Tris-HCl [pH 7.5], 10 mM NaCl, 3 mM MgCl_2_, 0.3% NP-40, 1X complete protease inhibitor cocktail (Roche, Switzerland) and 10% glycerol). After a 10-minute incubation on ice and a brief vortex of the sample, the lysate was centrifuged for eight minutes, 800 x *g* at 4˚C. The supernatant was separated and saved as the cytoplasmic fraction. The remaining nuclear pellet was washed by resuspending in 200 µL hypotonic lysis buffer and centrifuged for two minutes at 300 x *g*, this wash was added to cytoplasmic fraction. The nuclear pellet was lysed in 600 µL RLT buffer (RNAeasy kit, Qiagen) and 1.2 mL hypotonic lysis buffer was added. 600 µL RLT buffer was added to the cytoplasmic fraction. 1.8 mL 70% EtOH was added to each fraction and RNA was purified using the RNAeasy kit with on-column DNase digestion (Qiagen). qPCR was performed as previously described to probe location of DUBR, *GAPDH* mRNA (cytoplasmic control) and TERC ncRNA (nuclear control).

### Cell proliferation assays

The viability of cells were determined by modified microculture tetrazolium (MTT) assay (University at Buffalo, State University of New York, NY, USA) following the manufacturer’s suggested protocol. Briefly, 1.5 × 10^4^ cells were seeded in a 96-well flat-bottomed plate and left to grow overnight. The next day cells were treated with GapmeR and incubated for 24, 48 or72 hours. For MTT assay, growth medium was replaced with the working MTT solution following the manufacturer’s instructions. The resulting solution was incubated for another 20 min. After removing the MTT solution by aspiration, formazan crystals were dissolved in 100 mL of DMSO. The plates were then analyzed on the Synergy HTX Multi-Mode Microplate Reader (Agilent, USA) at 550 nm.

### Colony formation assay

5 × 10^4^ HCT116 cells were seeded in a 24-well plate and incubated overnight, followed by treatment with DUBR 1 or control GapmeR. After a 48-hour incubation, transfected cells were collected using trypsin (Corning) and resuspended in 10 mL McCoy’s 5 A modified medium. Then, 20 µL of the resuspended cell solution was added to wells of a 12-well plate containing 2 mL McCoy’s 5 A modified medium and incubated for 10 days. Finally, the medium was discarded, and the cells were washed in 1 mL 1X PBS, fixed using 1 mL of fixation buffer (3:1 ratio of methanol to acetic acid) and stained with 0.1% crystal violet (Ricca Chemical, USA). The number of colonies in each well was counted by hand. Colony area % was calculated using the ImageJ plugin ColonyArea (Guzmán et al. 2014).

### Scratch assay

5 × 10^4^ cells were seeded in a 24-well plate and incubated overnight, followed by treatment with DUBR 1 or control GapmeR. After a 48-hour incubation, a 20 µL pipette tip was used to remove cells in a line and the media was removed and replaced. The wells were imaged at 2-, 7- and 24-hours post scratch. Images were analyzed and % area covered was calculated using ImageJ.

### Cell cycle analysis by flow cytometry with propidium iodide (PI) staining

5 × 10^4^ HCT116 cells were seeded in a 24-well plate and incubated overnight, followed by treatment with DUBR 2 or control GapmeR. After a 48-hour incubation, transfected cells were collected using trypsin (Corning) and washed twice with 1X PBS. Cells were then permeabilized and fixed with 70% ice-cold ethanol drop-wise with gentle vortexing to prevent clumping and incubated for 15 min on ice. Cells were washed twice with 1X PBS. Then, cells were resuspended in PI staining solution (3.8 mM sodium citrate, 50 µg/mL PI (Biotium, USA), 40 µg/mL RNase A (Zymo Research, USA)) and incubated for 40 min at 37˚C in the dark. Cells were centrifuged and the supernatant removed. Finally, the cells were resuspended in 500 mL ice cold 1X PBS and examined on the S3e Cell Sorter (Bio-Rad Laboratories, USA). The data was analyzed using FlowJo software (BD Biosciences, USA).

### RNA antisense purification with mass spectrometry (RAP-MS)

1 × 10^7^ HCT116 were used for each RAP-MS experiment and performed as previously described (Trang et al. 2023). LC-MS/MS analysis was done at Sanford Burnham Prebys Proteomics Core on a Thermo QExactive instrument. Mass spectrometry datasets as well as MaxQuant peptide search results and parameter files are available via ProteomeXchange with identifier PXD057811.

DUBR RAP-MS hits were filtered for proteins with at least two peptides and not found in snRNA U1 and luciferase mRNA RAP-MS hits. This list was then checked for non-nuclear localized proteins using UniProt website which were removed. The remaining proteins were clustered using STRING analysis (Szklarczyk et al. 2023).

### RNA immunoprecipitation (RIP)

Native RNA immunoprecipitations were performed following previously published methods (Gagliardi and Matarazzo 2016). Briefly, 1 × 10^7^ HCT116 cells were harvested for each RIP and resuspended in 110 µL of cold polysome lysis buffer (20 mM Tris-HCl [pH 7.5], 50 mM KCl, 10 mM MgCl_2_, 1 mM DTT, 1X Complete protease inhibitor cocktail (Roche), 200 units/mL RNase inhibitor murine (New England Biolabs). Cells were lysed by incubation at 4˚C for 5 min followed by incubation at -80˚C for 2 h before centrifuging for 10 min at 20,000 x *g*. The supernatant was collected. For each RIP 75 µL of Dynabeads Protein G (Thermo Fisher Scientific) were washed twice with NT-2 buffer (50 mM Tris-HCl [pH7.5], 150 mM NaCl, 1 mM MgCl_2_, 0.05% NP-40) and resuspended in 100 µL NT-2 buffer, with 5 µg of appropriate antibody: IgG as a negative control (10284-1-AP, Proteintech), DNMT1 (NB100-56519, Novus Biologicals, USA), CHD4 (A11574, Abcam), HDAC1 (sc-81598, Santa Cruz Biotechnology), HDAC2 (A2084, Abcam). The beads/antibody mixtures were incubated for 60 min at room temperature, then beads were washed six times with NT-2 buffer before resuspending in NET-2 buffer (50 mM Tris-HCl [pH 7.5], 150 mM NaCl, 1 mM MgCl_2_, 0.05% NP-40, 20 mM EDTA [pH 8.0], 1 mM DTT, 200 units/mL RNase inhibitor murine). Each antibody had 1 × 10^7^ cells worth of lysate added and the total volume brought to 1 mL with NET-2 buffer. All samples were incubated overnight at 4˚C with gentle rotation, followed by six washes with NT-2 buffer. To elute RNA, the beads were resuspended in 18 µL NLS elution buffer (20 mM Tris–HCl [pH 8.0], 10 mM EDTA [pH 8.0], 2% NLS, and 2.5 mM TCEP), heated at 95˚C for 5 min and then incubated with 2 µL of Proteinase K (Thermo Fisher Scientific) for 1 h at 50˚C to digest proteins, then the beads were separated and standard RNA silane cleanup with Dynabeads MyOne Silane (Thermo Fisher Scientific) was performed on the supernatant before reverse transcribing the RNA using SuperScript III to perform qPCR.

### Native H3K27ac chromatin immunoprecipitation with qPCR (ChIP-qPCR)

HCT116 cells were treated with control or DUBR 1 GapmeR, and after 48 h, cells were trypsinized and pelleted. ChIP was performed based on the Alonso et al. 2018 protocol (Alonso et al. 2018) with the following alterations: (1) All buffers were supplemented with 5 mM sodium butyrate, (2) per ChIP, 10 µg of chromatin was used with 0.5 µg of antibody (AB177178, Abcam) and 25 µL of Dynabeads Protein G (Thermo Fisher Scientific), and (3) qPCR was performed as described in above methods section.

### Assay for transposase-accessible chromatin with sequencing (ATAC-seq)

HCT116 cells were treated with control or DUBR 1 GapmeR for 48 h in triplicate. ATAC-seq sample preparation was performed using the Active Motif ATAC-seq Kit (53150, Active Motif, USA) following provided protocol. DNA-seq libraries were pooled and then sent to the UCSD Institution for Genomic Medicine where the quality of the libraries was examined by Agilent BioAnalyzer, and sequencing was performed on a NovaSeq X Plus 10B platform with the run type of PE100. ATAC-seq data are available on the NCBI Sequence Read Archive associated with the BioProject PRJNA1186227.

Sequencing quality was checked with FASTQC v0.12.1 (Andrew, n.d.). Sequences with transposon adapters identified by FASTQC were trimmed and remaining reads longer than 20 nucleotides were kept. Filtered sequences were aligned with STAR (Dobin et al. 2013) to human reference genome assembly GRCh38 (Schneider et al. 2017) and GENECODE gene annotation release 38 (Frankish et al. 2019). Reads aligning to the mitochondrial chromosome, unknown chromosomes, or unpaired reads were removed with samtools version 1.3.1 (Danecek et al. 2021). Samtools was also used to fix mates, sort, remove duplicate reads (pairs with identical read start and ends) and subsample based on remaining unique reads.

Peak calling was based on method 4 from published ATAC-seq normalization and peak calling pipeline (Reske et al. 2020). Processed BAM files were filtered to remove blacklisted regions (Amemiya et al. 2019). Remaining reads were split into 300-nucleotide regions and filtered for a greater read count than the surrounding 2 kb region, then peaks were called on each BAM file by csaw with loess-normalization (Lun and Smyth 2016). Peaks were compared for differential accessibility with edgeR (Robinson et al. 2010) and filtered for an FDR < 0.05.

The activity-by-contact model (Fulco et al. 2019) was used to associate enhancers with genes in HCT116 cells using publicly available H3K27ac ChIP-seq (ENCFF176BXC) (ENCODE Project Consortium 2012), Hi-C data (ENCSR477GZK) (Du et al. 2021) and ATAC-seq (ENCFF927YUB) (ENCODE Project Consortium 2012). Resulting enhancers-gene pairs were intersected with ATAC-seq differentially accessible peaks and RNA-seq differentially expressed genes using bedtools version 2.30.0 (Quinlan and Hall 2010).

### Illumina RNA sequencing

HCT116 cells were treated with control or DUBR 1 GapmeR for 48 h. RNA was isolated with RNeasy kit (Qiagen) and purified using RNA Clean & Concentrator kit (Zymo). Illumina RNA-seq libraries were prepared at the UCSD Institution for Genomic Medicine using Illumina stranded mRNA prep after poly-A selection. The quality of the libraries was examined by Agilent BioAnalyzer, and sequencing was performed on a NovaSeq S4 with the run type of PE150. Raw paired-end FASTQ sequencing reads were mapped with STAR version 2.7.9a (Dobin et al. 2013) to human reference genome assembly GRCh38 (Schneider et al. 2017) and GENECODE gene annotation release 38. FeatureCounts version 2.0.3 (Liao et al. 2014) was used to generate read count matrices, and DESeq2 version 1.34.0 (Love et al. 2014) analysis identified differential expression of RNA transcripts between the triplicate samples of control or DUBR 1 GapmeR treated cells. Gene ontology enrichment analysis was performed with GOnet (Pomaznoy et al. 2018). RNA sequencing data are available on the NCBI Sequence Read Archive associated with the BioProject PRJNA1186227.

## Data Availability

The datasets produced in this study are available in the following databases: RNA-seq and ATAC-seq data: Illumina high-throughput sequencing run data are available on the NCBI Sequence Read Archive associated with the BioProject PRJNA1186227. Proteomics data: raw mass spectrometry data and MaxQuant peptide search results are available at ProteomeXchange with identifier PXD057811. All unique and stable reagents generated in this study are available from the Lead Contact with a completed Materials Transfer Agreement.
